# The evidence for repurposing anti-epileptic drugs to target cancer

**DOI:** 10.1007/s11033-023-08568-1

**Published:** 2023-07-07

**Authors:** Mir Aroosa, Jonaid Ahmad Malik, Sakeel Ahmed, Onur Bender, Nafees Ahemad, Sirajudheen Anwar

**Affiliations:** 1 Department of Pharmacology, Jamia Hamdard, New Delhi, India; 2 Department of Biomedical Engineering, Indian Institute of Technology Ropar, Rupnagar, Punjab India; 3 Department of Biomedical Engineering, Indian Institute of Technology (IIT), Ropar, Ropar, India; 4 Department of Pharmacology and Toxicology, National Institute of Pharmaceutical Education and Research, Ahmedabad, Gujarat India; 5 Biotechnology Institute, Ankara University, Ankara, Turkey; 6 School of Pharmacy, Monash University Malaysia, Jalan lagoon selatan, Petaling Jaya, Selangor, DE Malaysia; 7 Department of Pharmacology and Toxicology, College of Pharmacy, University of Hail, Hail, Saudi Arabia

**Keywords:** Breast cancer, Antiepileptic drugs *for cancer*, Drug repurposing, Cancer treatment, Antiepileptic drugs

## Abstract

**Abstract:**

Antiepileptic drugs are versatile drugs with the potential to be used in functional drug formulations with drug repurposing approaches. In the present review, we investigated the anticancer properties of antiepileptic drugs and interlinked cancer and epileptic pathways. Our focus was primarily on those drugs that have entered clinical trials with positive results and those that provided good results in preclinical studies. Many contributing factors make cancer therapy fail, like drug resistance, tumor heterogeneity, and cost; exploring all alternatives for efficient treatment is important. It is crucial to find new drug targets to find out new antitumor molecules from the already clinically validated and approved drugs utilizing drug repurposing methods. The advancements in genomics, proteomics, and other computational approaches speed up drug repurposing. This review summarizes the potential of antiepileptic drugs in different cancers and tumor progression in the brain. Valproic acid, oxcarbazepine, lacosamide, lamotrigine, and levetiracetam are the drugs that showed potential beneficial outcomes against different cancers. Antiepileptic drugs might be a good option for adjuvant cancer therapy, but there is a need to investigate further their efficacy in cancer therapy clinical trials.

**Graphical Abstract:**

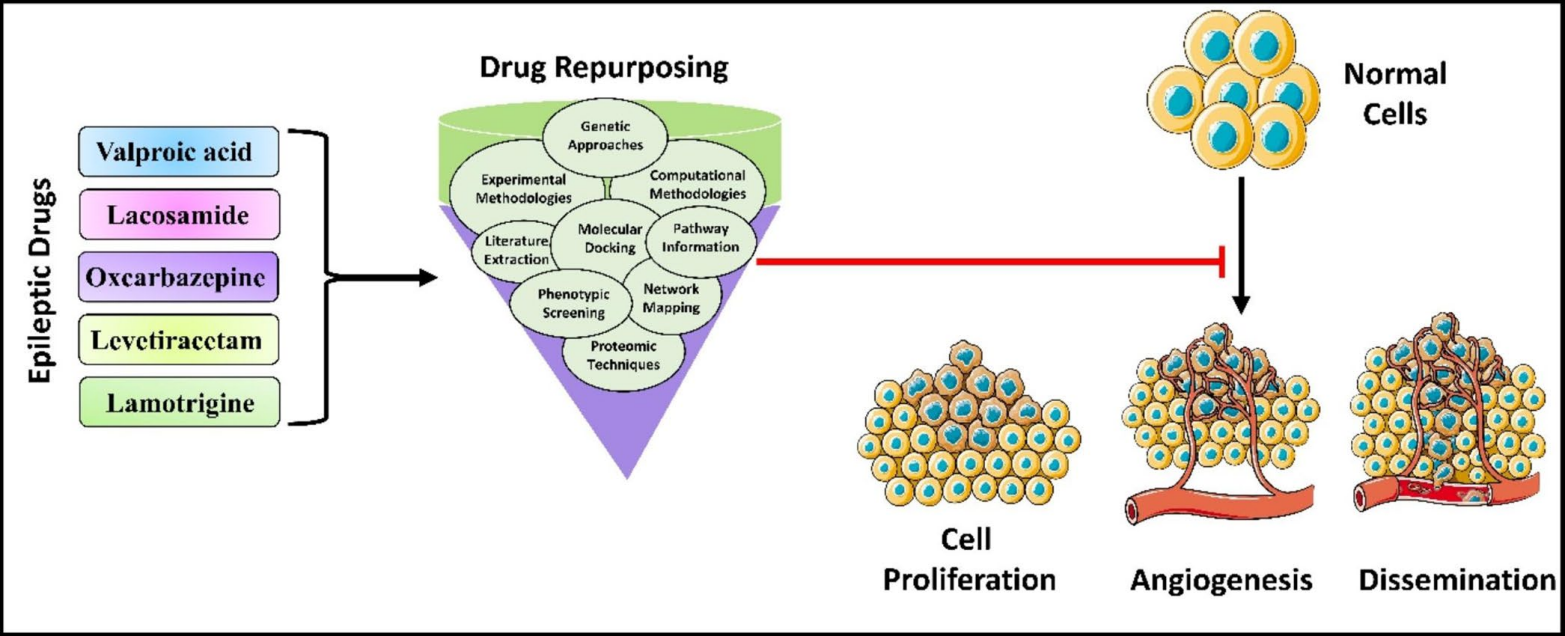

## Introduction

Cancer is among the leading reasons of death globally. Increasing understanding of human neoplastic illnesses and technological advancements make it possible to develop novel antineoplastic agents to reduce cancerous deaths [1–4]. A recent example that completely changed cancer perception is PD1, blocked by dostarlimab [5]. Drug development requires a long period for exploration and manufacture, with medications requiring up to 15 years to enter the therapeutic or clinical space and financing from corporate or scientific organizations [6]. Screening the safety and efficacy of drugs in human participants in a clinical trial (CT) [7].

**Fig. 1:**
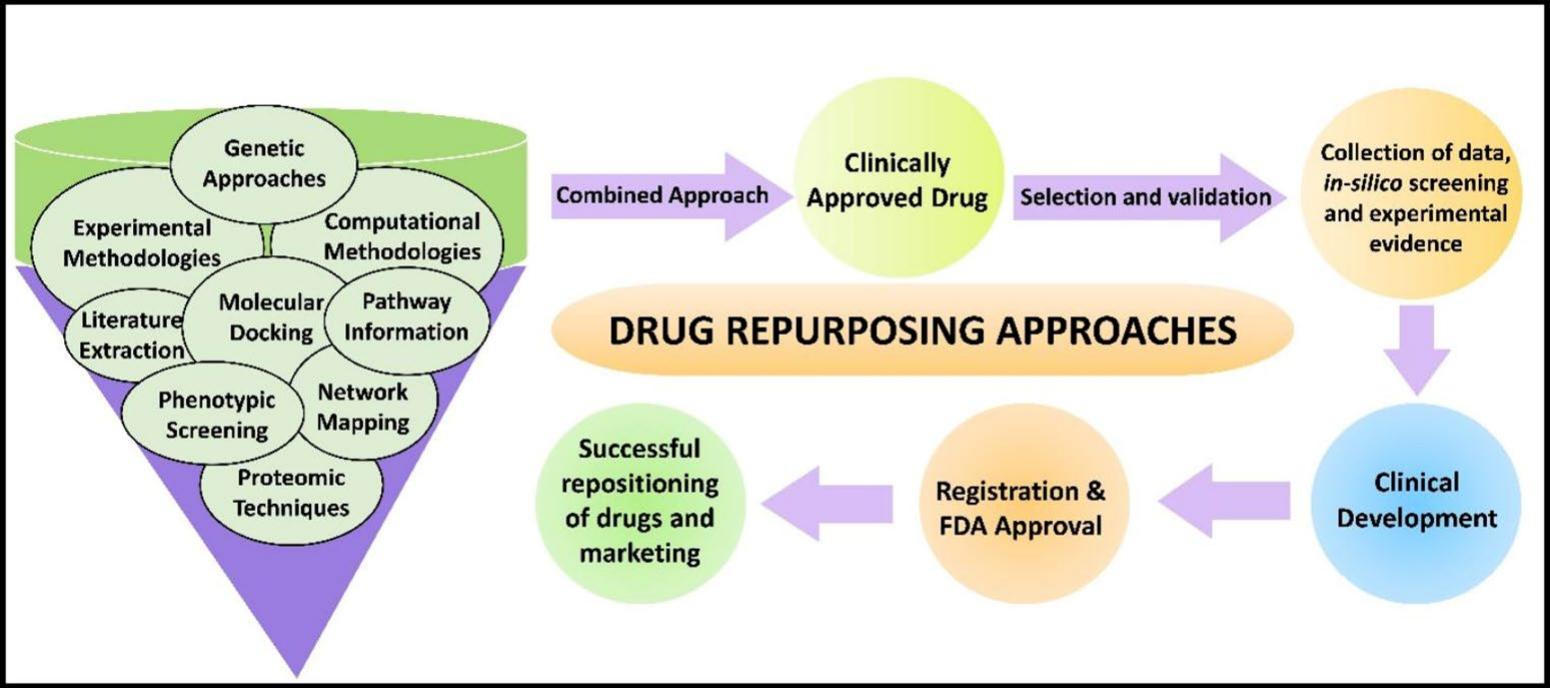
Demonstrates the required approaches toward drug repurposing: An overview of various approaches required for drug repurposing to target cancer. The combined approaches with proper selection and validation studies like data collection, in-silico, and experimental shreds of evidence help in clinical development following registration and FDA approval for the successful repositioning of drugs and marketing

Furthermore, most innovative drugs/molecules failed to institute safety and efficacy in clinical trial and, as a result, failed to access the therapeutic or clinical space: the rate of success is < 10%. Many organizations are reanalyzing commercially licensed pharmaceuticals from a drug repurposing standpoint as a unique strategy for overcoming these constraints [6]. Drug repositioning (“creative innovations for old medications”) is a strategy to explore new indications for approved or experimental drugs that go beyond the primary medical indication [8, 9]. The main advantages of this technique are that the pharmacodynamic, pharmacokinetic, and toxicity profiles of medications have been extensively documented in preclinical and Phase-1 trials. Hence, these medications might promptly advance through Phase-2/3 of clinical trials, and developmental costs could be greatly lowered. Thus, drug repurposing can result in a lower risky business model with reduced developmental costs, notably if new drug failures during research and development are avoided (Fig. 1) [3, 10, 11]. The first productive descriptions of drug repurposing were primarily the consequence of coincidental innovations.

In contrast, resultant comprehensive methodologies for identifying non-oncology drugs that could presumably be repurposed in cancer therapy originated (Fig. 1). They can be divided into computational and experimental methodologies [12]. Computational ones depend on high throughput screening and bioinformatics tools such as molecular docking or network mapping.

In contrast, experimental depends on assays that are based on activity such as proteomic techniques or chemical genetic approaches to recognize pertinent relationships between novel targets and defined drugs, or cell-based phenotypic screening focused on the classification of prevalent phenotypic criteria (e.g., proliferation, exosome biogenesis modulation, cell cycle profiling) without previous knowledge of the target [13]. Several applicant drugs have been under investigation, from non-cancer to cancer therapy. Some examples include celecoxib, primarily used for arthritis treatment, which is under investigation for lung, colorectal, and breast cancers (NCT01695226); aspirin in colorectal cancer; valproic acid, which is an antiepileptic drug that has been under investigation for leukemia (NCT00530907); metformin, an anti-diabetic drug is under investigation for breast, prostate and colorectal cancers (NCT00897884), angiotensin receptor blockers, which include losartan, primarily used for the treatment of hypertension, has been under investigation for breast and pancreatic cancer (NCT01821729) [14]. Disulfiram, initially authorized as an anti-alcoholism drug, has displayed antitumor effects in many preclinical studies, most recently on several types of human cancer such as; lung, breast, prostate, pancreatic, and melanomas. Moreover, has a viable advancement in the intervention of non-small cell lung cancer and glioblastoma [15]. All drugs employed in clinical treatment can approach multiple targets [16, 17]. As a result, if the targets of these medications are extremely consistent with cancer, there is a good chance that those with similar targets will be therapeutic for additional cancer patients. Historically, drug repurposing has been mostly opportunistic and fortuitous [18, 19]. It is worth mentioning that the repurposing strategy necessitates the systematic assimilation of research data from various disciplines, including synthetic chemistry, in silico modeling, systems pharmacological approaches, in vitro screens, clinical studies, and in vitro and in vivo functional assays [20]. As evidenced by a huge body of data through in vitro and in vivo investigations or clinical trials, several novels recognized non-oncology medications repurposed for cancer therapy function by suppressing proliferation and promoting cell death.

Furthermore, formerly employed for other conditions, these medications have robust drug safety data and are frequently affordable (particularly if accessible in their generic form) [21]. In some circumstances, a drug’s pharmacological activity results from blocking specified targets, off-targets, or a hybrid of both. This tendency is known as “polypharmacology,“ originally characterized as a compound’s bonding capacity to many targets [22], which encourages exploring the further indications of already approved drugs. The main research question was to discover the anticancer properties of these drugs and the interlink pathways of cancer and epilepsy. This review has highlighted various findings that can help repurpose the treatment of multiple types of cancer. We have focused on various drugs that have entered clinical trials with positive results and others that have depicted good results in preclinical studies.

## Challenges with the existing therapies

Drug resistance is the primary obstruction to treating cancer patients. The primary approach to overcome the resistance is using a combination therapy of drugs with non-overlapping modes of action or polychemotherapy [2–4, 23, 24]. This pragmatic strategy was quite effective in some types of lymphoma, breast, and testicular cancer [25, 26]. As a result, combined chemotherapeutic approaches emerged as a new perspective for cancer therapy, resulting in a complicated regimen. Furthermore, various dose intensity techniques, such as shorter-interval infusions of chemotherapy or high doses of chemotherapeutic interventions with growth factor stimulation to prevent prolonged bone marrow depression, contributed to the better efficacy of these therapies by inhibiting early tumor re-growth [27, 28]. Polychemotherapy’s accomplishments had plateaued by the turn of the century, some 50 years after its commencement. Surgery, radiation, and polychemotherapy were insufficient to cure many cancers [26]. These cause cancer cell inhibition and offer targeted and intelligent treatment options.

Consequently, novel treatment techniques to tackle the key enabling features and acquired capacities that turn healthy cells and tissues into cancers have begun to emerge. Introducing medicines that disturbed these distinguishing traits, such as targeted therapy, was a significant step forward. Indeed, greater comprehension of cancer biology drivers has evolved into highly effective medicines targeting nuclear receptors, tyrosine kinases, and other molecular targets. Following the early success of androgen receptor (AR) antagonists and epidermal receptor and BCR-ABL, HER2, and EGFR inhibitors, a great effort was launched to create medicines that target oncogenes and other critical cellular liabilities.

Oncological therapy has progressed by employing immunological techniques to recognize and attack cancer. Anti-PD-1/PD-L1 and anti-CTLA4 monoclonal antibodies that inhibit negative regulators of the adaptive immune system, or checkpoints, have generated significant antitumor activity and even cures in various ways tumor types [29–33]. Nonetheless, as it was formerly found with standard chemotherapy, subsequent resistance to targeted and immunological treatments is the expected norm [26]. According to statistics, drug resistance is responsible for more than 90% of deaths in cancer patients. Multidrug resistance (MDR) in cancerous cells undergoing chemotherapeutic treatment can be attributed to several processes, notably increased drug efflux, genetic variables (Mutations, gene rearrangements, epigenetic modifications), enhanced DNA repair capability and growth factors, and heightened xenobiotic metabolism. These mechanisms reduce the treatment effectiveness of given medications, making tumor treatment more challenging. Since cancer is a heterogeneous and multi-targeted disease, this approach is crucial for success in combating it.

The presently available anticancer drugs have many challenges, including drug resistance, side effects, cost, less efficacy, less potency, or non-responsiveness [3, 4, 25]. Cancer poses so many different challenges than any existing disease. The most potent anticancer drugs available today are their non-specificity towards cancer cells, like cisplatin, doxorubicin, etc., which make cytotoxic to normal cells leading to tissue damage [25]. The most important challenge is that these anticancer drugs are administered intravenously, making them more cytotoxic [34]. The anticancer drugs aimed to target the tumor sites affect the whole body leading to ineffectiveness against targeted cancer cells. The issue is that anticancer drugs like vincristine and vinblastine from the natural source have challenges of drug solubility, dosage, yield, sufficient delivery, and bioavailability [35]. The chemical stability of anticancer drugs is another challenge that affects the drug’s potency and the drug uptake and activity in the tumor sites, leading to obstruction of the dose-effect [25]. The other challenges are discussed below.

## Mechanisms of drug resistance

### Drug efflux enhancement

#### P-Glycoproteins

Increased chemotherapeutic drug efflux from cancer cells results in lesser drug accumulation. MDR is the most common cause of chemotherapeutic drug resistance. Drug efflux transporters, or efflux pumps, are primarily accountable for MDR [36]. ATP binding cassette subfamily B member 1/P-glycoprotein (ABCB1/P-gp), or breast cancer resistance protein (BCRP), is an ABC protein found on the cell membrane that regulates the absorption, metabolism, distribution and excretion of various chemical substances. Since these proteins guard cells against apoptosis caused by elevated intracellular drug concentrations, they can also impede the administration of drugs by reducing bioavailability, intracellular concentration, and BBB transition. P-gp, extensively expressed on the endothelial cell membrane, leads to limited chemotherapeutic drug administration in specific areas, particularly in treating brain tumors, where anticancer medicines are often inefficient in passing through the BBB [37]. The size of the tumor is also important for drug penetration. Chemotherapeutic drugs are typically less effective in large tumors because of the low blood supply than in tiny tumors with practically free oxygen and nutrition supply exposure. The P-gp safeguards the brain from potentially harmful substances while limiting access to therapeutic medicines that are accountable for the greater intricacy of the therapy. Most of the time, the only option to get around the barrier is to elevate the quantity of the medicine, which typically results in general toxicity. That is why increased drug efflux has been identified as one of the primary mechanisms of tumor cell resistance to chemotherapeutics [38, 39]. MDR occurs when P-gp is overexpressed on cancer cells. P-gp’s transporter structure contains several drug bindings sites that engage with various chemotherapeutic medicines, including etoposide, paclitaxel, doxorubicin, and many others.

**Fig. 2:**
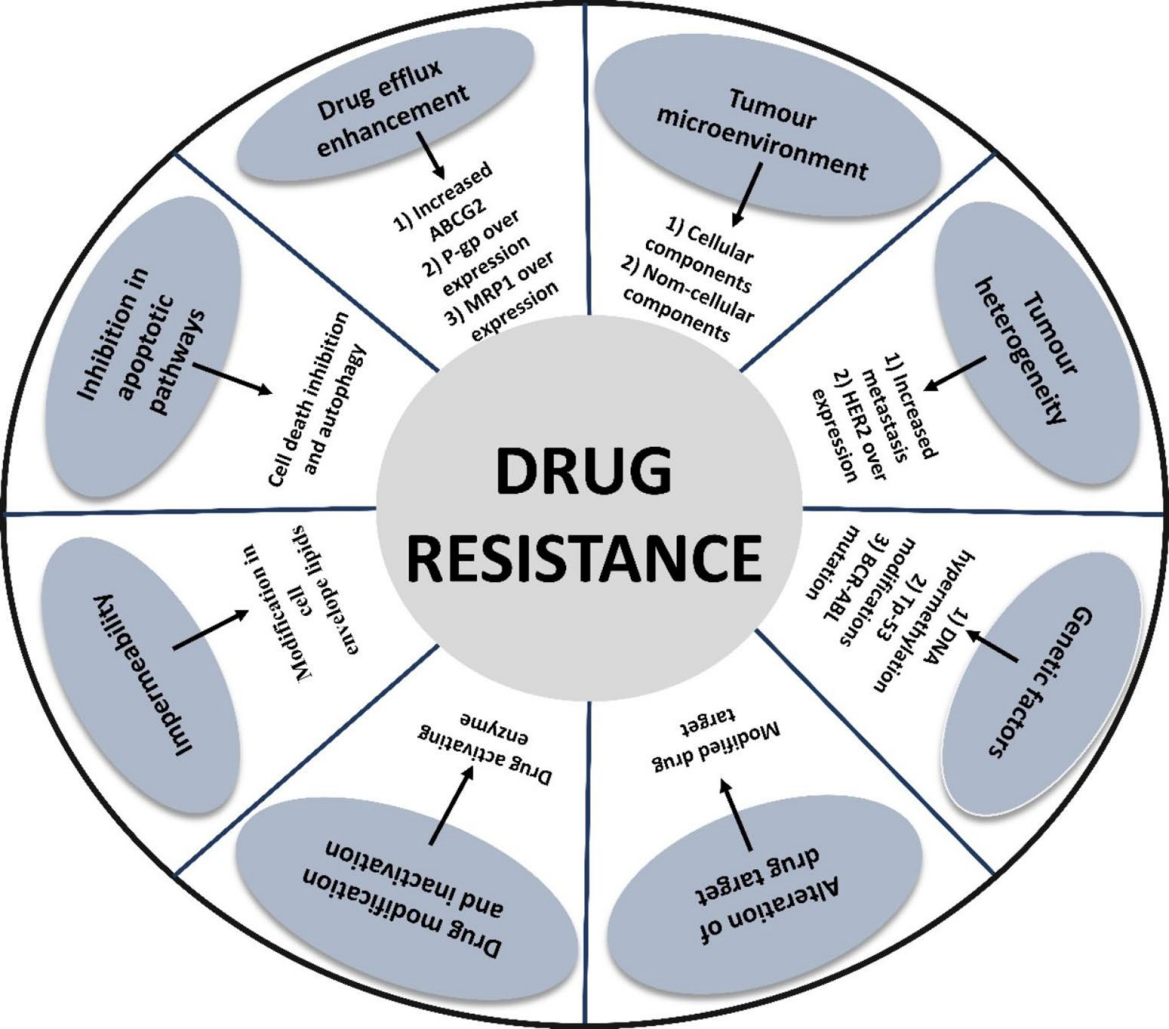
An overview of several contributing factors responsible for drug resistance in cancer. Most importantly, these factors act via different mechanistic pathways like drug modification and inactivation, alteration of drug targets, genetic factors, tumor heterogeneity, tumor microenvironment, drug efflux pumps, and inhibition of apoptotic pathways. All these factors are responsible for cancer drug resistance

Cancers of the liver, colon, adrenal gland, pancreas, and kidney have the top levels of P-gp expression, whereas soft tissue cancers, neuroblastoma, and hematological malignancies have moderate amounts. P-gp levels are initially low in breast, ovarian, oesophageal, and lung malignancies. However, the levels of P-gp efflux transporters rise when the tumor develops resistance to chemotherapeutic treatments [36, 40]. First-generation P-gp inhibitors include trifluoperazine, quinidine, cyclosporine-A, reserpine, verapamil, tamoxifen, yohimbine, and vincristine [41, 42]. ABCB1 overexpression has been linked to chemotherapeutic failure (Fig. 2). Furthermore, MDR murine melanoma cells have significant ABCB1 gene amplification. The biological basis for “MDR” p-glycoprotein’s characteristic feature is its broad substrate selectivity, including vinca alkaloids, anthracyclines, and epipodophyllotoxins [43].

#### Cancer resistance protein (CRP)

ABCG2, one constituent of the extensive ABC superfamily, was overexpressed in adriamycin-resistant human-derived breast cancer cells. Additionally, the hypoxic state has been shown to influence ABCG2 expression. Because of increased ABCG2 expression, cancer cells or stem cells in hypoxic conditions resist medicines [44]. The ABCG2 gene codes for BCRP. It was discovered in a drug-resistant human breast cancer cell line exposed to mitoxantrone and tariquidar, both P-gp inhibitors [45].

Multidrug resistance-associated proteins (MRP) is a component of the mammalian ABC family of biological membrane transporters known to produce MDR. This transporter was identified while working on the H69AR cell line, a drug-resistant small-cell lung cancer [46]. MRP1 overexpression has been linked to anticancer drug resistance. The quantity of decreased GSH is required for unmodified chemotherapeutic drugs to be transported by MRP1 [47].

### Cellular and non‑cellular factors in the context of the tumor microenvironment

The interconnections involving cancer cells and nearby tumor microenvironment (TME) components cause TME-mediated innate resistance at the time involving chemotherapy. The interactions between cancer cells and nearby TME components cause TME-mediated innate resistance during cancer treatment. This established resistance given by the TME appears to be a host compensatory reaction to pharmacologic exposures. TME’s cellular (fibroblasts, endothelial cells, immune cells) and non-cellular components (oxygenation, soluble substances such as cytokines, extracellular matrix, pH, vascular endothelial growth factor (VEGF)) contribute to drug resistance (Fig. 2) [48, 49]. In the particular instance of lung, breast, and prostate cancer, for example, IL-6 can significantly raise drug resistance by blocking apoptosis via stimulation of Janus kinases (JAK)/signal transducer and activator of transcription 3 (STAT3), phosphatidylinositol-3 kinase (PI3K)/protein kinase B (Akt), and Ras-MAPK pathways. Due to the hypoxic situation, the relatively low pH, changing oxygen levels, and increased reactive oxygen species (ROS) levels encourage angiogenesis, metastasis, tumor severity, and an elevation in MDR proteins, reducing the treatment effectiveness of chemotherapy drugs [50]. It was reported that efflux of anticancer medicines after encapsulating them in exosomes. They discovered a link between drug efflux and drug sensitivity in many tumor models and suggested that exosomes shed with drug resistance [48].

#### Tumour heterogeneity

A research group observed that the Kirsten rat sarcoma (KRAS) mutation was found to be the main reason for the resistance in esophagogastric cancer, so this synchronously augments epidermal growth factor receptor (EGFR) and human epidermal growth factor receptor (HER2), and it was demonstrated in roughly 50% of the mesenchymal-epithelial transition (MET)- exacerbated esophagogastric cancer in patients. The T790M EGFR mutation is a genetic foundation of acquired tyrosine kinase inhibitor (TKI) resistance. A change in KRAS/MAPK signaling or a BRAF or KRAS gene mutation causes drug resistance in MEK1/2 inhibitors in cancer cells like colorectal cancer, ovarian cancer, and others [51, 52]. Heterogeneity is a distinguishing trait of tumor cells in comparison to healthy ones. Cells are characterized by many phenotypic and morphological aspects that comprise gene expression, cellular morphology, epigenetics, metabolism, motility, proliferation, transcriptome, and metastatic potential. Intertumoral heterogeneity relates to the diversity among patients with identical histology but differing genetic differences, somatic mutation, and environmental variables, while intra-tumoral heterogeneity relates to variability inside the tumor. Intra-tumoral heterogeneity is a significant contributor to the deadly implications of cancer due to drug resistance. As a result, it is accountable for therapeutic failures and may be a non-heritable and heritable driver of variation. Cancer stem cells (CSCs) are thought to persist even after being formed predominantly from an organ’s normal stem cells. A research group has published a detailed study of CSCs in drug resistance, including their therapeutic options for overcoming resistance as a subset of cells in a tumor microenvironment [48, 53].

### Genetic alteration

Genetic alterations, widely detected in tumor cells, are considered one of the primary culprits of chemotherapeutic drug failure. Modifications in the TP53 gene, typically found in tumor cells, are one of the most well-known indicators of tumorigenesis. BCR-ABL tyrosine-kinase antagonists, like imatinib, popularly used as the drug of choice in patients with chronic myeloid leukemia (CML), inhibits ATP binding to the BCR-ABL kinase receptor, hence leading to apoptosis in tumor cells. According to the data, alterations in the BCR-ABL gene, which is connected with the drug-binding area, frequently result in imatinib resistance during CML chemotherapy [54, 55]. Topoisomerase-II targeted medicines, such as etoposide, are commonly used to suppress replication by inhibiting the expression of this enzyme. However, topoisomerase gene alterations modify its nuclear localization, resulting in resistance to tumor cells.

Furthermore, these medications are not exclusive to cancerous cells; instead, they interfere with the whole genome, severely limiting their safe use in managing cancer [56]. The most recent research underlines the critical significance of epigenetic changes in tumor cells in chemotherapeutic treatment resistance. Cancer development could be influenced by tumor suppressor gene silencing via DNA hypermethylation or oncogene expression enhancement via DNA hypomethylation. The epigenome undergoes numerous modifications throughout carcinogenesis, including genome-wide loss of DNA methylation, localized hypermethylation (particularly in CpG promoter islands of tumor suppressor genes), and worldwide alteration in histone modifications, and modifications in miRNA expression [57, 58]. Healthy cells restore damage caused to DNA or undergo apoptosis; however, cancerous cells overcome the strict control mechanism and modify DNA repair. Some chemotherapeutic medications, such as platinum, actively cause DNA damage, while others, such as Irinotecan, doxorubicin, and others, degrade the DNA implicitly through topoisomerase enzyme inhibition. The ability of some tumor cells to restore DNA damage affects the efficacy of chemotherapeutic drugs [59, 60].

## Cross-talk between oncogenic and epileptogenic pathways

Although the functions of the process involved in inflammation in epilepsy have recently been discovered, it has been historically recognized that cancer and inflammation progress simultaneously. A tumor induces an inflammatory reaction and vice versa. Tumor cells modulate the expression of chemokines and cytokines, assisting in the recruitment of inflammatory cells and supporting tumor expansion. In contrast, glutamate is released in inflamed regions surrounding the expanding mass that stimulates multiple number of cells, promoting an inflammatory micro-environment and thereby increasing DNA oxidative damage and the stimulation of both epigenetic and genetic alterations. These modifications affect cellular signaling pathways that control proliferation, survival, and invasions [61, 62]. Hyperactivation of specialized receptor subclass of glutamates (GluRs) like ionotropic NMDA, AMPA receptor, and metabotropic mGluR1-8 combined with impeded GABAergic inhibition due to a reduction in numerous GABA-A (Gamma-aminobutyric acid) subtypes and the decreased expression of its receptor like GABA-AR (GABA-Androgen receptor) suggests notable hyperexcitability of neurons that leads to spontaneous seizures [63].

Moreover, the same disbalance among excitatory and inhibitory receptors appears to have a part in the formation of tumor lesions. Multiple investigations have established that glutamate and the previously stated GluRs may play a role in tumorigenesis and invasion in non-neural and neural tumors [64, 65]. Dysfunction in the metabolism of GABA could be a symptom of a cell’s defensive response during tumorigenesis, given the complex disparity between activating and inhibiting AA in brain tumor tissues. GABA has been identified as a significant down regulator of proliferation in stem cells. Analysis of GABA binding sites in glioblastomas revealed that those greater malignancies were linked to lesser GABA binding [66].

On the contrary, the significance of androgen receptor GABA-AR overexpression in breast cancer cerebral metastasis is unknown. However, it might be a malignant modification essential for brain colonization [67]. Interestingly, in contrast to GABA-AR amplification, breast cancer-lead cerebral metastasis stimulates GABA transporter and GABA transaminase, allowing the cell to utilize GABA as a source of energy via the GABA shunt pathway [68, 69]. Furthermore, even operationally changed voltage-dependent ion channels in tumor cells could promote hyperactivity, tumor development, and metastasis [70, 71]. The importance of these membrane pathways in cellular proliferation has been thoroughly described in various cellular forms in the context of various cancerous cells. This engagement was first established for potassium channels and additional voltage-gated ion channels like calcium and chloride [72, 73]. For instance, potassium and calcium ion channel activity and expression modifications have been linked to decreased patient survival and more severe brain tumor behavior [63, 74].

Valproic acid has received more attention regarding the antiepileptics that seem to provide anticancer impact. In vivo/in vitro investigations revealed that valproic acid inhibits histone deacetylases (HDAC) in patients with glioblastoma. Furthermore, epigenetic alterations, including abnormal histone acetylation and DNA methylation, are widespread in malignancies, providing a compelling argument for using these antiepileptic drugs as an epigenetic combination treatment [75, 76]. Other potential treatment signs for antiepileptics as antitumor agents include levetiracetam as an O(6)-Methylguanine-DNA-methyltransferase (MGMT) transcription antagonist and brivaracetam and lacosamide for their anticarcinogenic and anti-migration ramifications due to the attenuation of specific microRNAs, including miR-107 and miR-195-5p [77, 78].

### Valproic acid

**Fig. 3:**
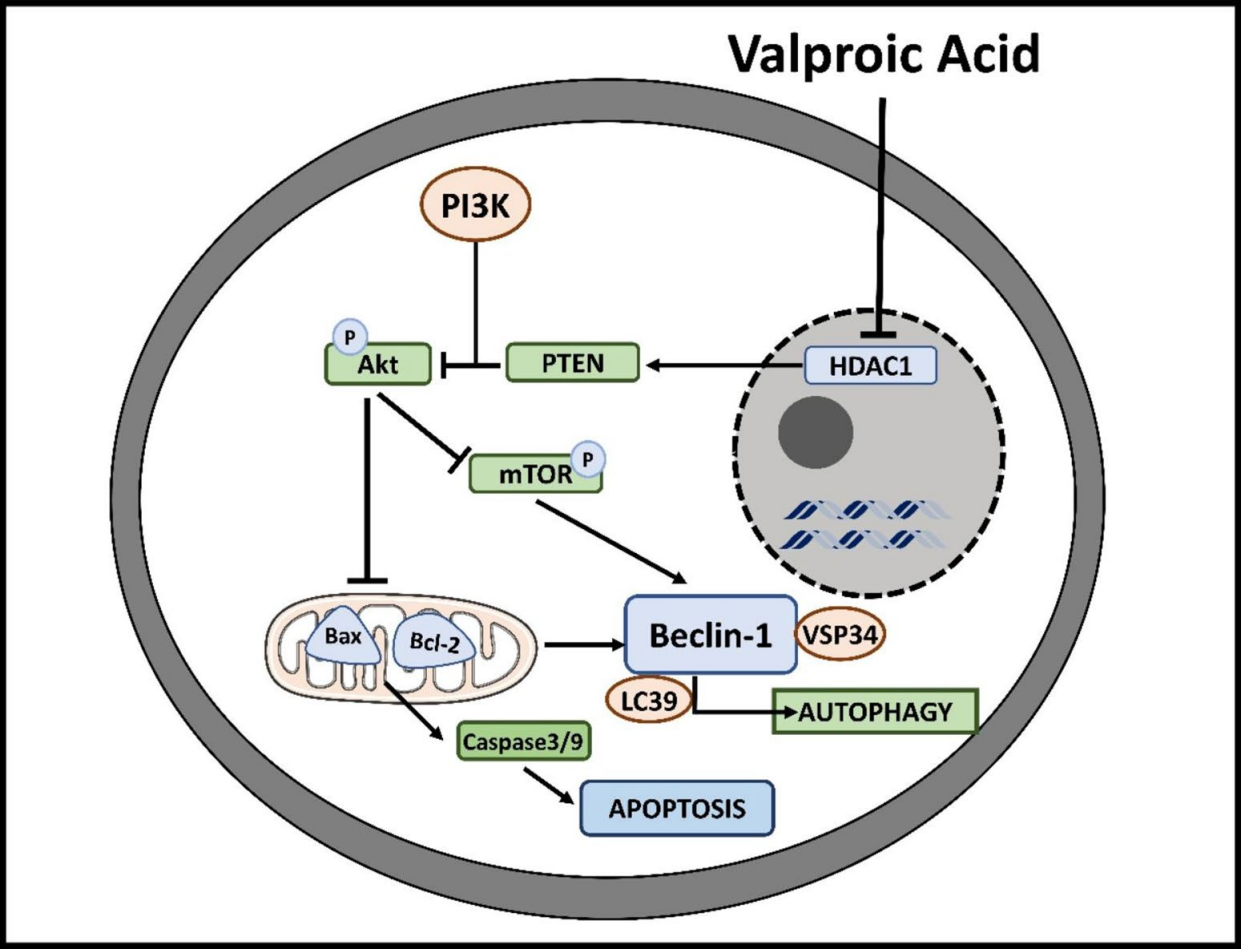
Mechanism of action of valproic acid. An overview of valproic acid’s mechanism blocks HDAC, which blocks the downstream PTEN/PI3K-Akt axis. This inhibition ultimately blocks mTOR and proteins Bax and Bcl-2, promoting caspase-3 and caspase9 expression and causing apoptosis and autophagy

Valproic acid promotes GABAergic functioning and decreases excitatory signals by post-down-regulation of voltage-gated Na^2+^ channels and NMDA glutamatergic receptors. Furthermore, valproic acid has also evolved as an antineoplastic medication. Valproic acid suppresses tumor gene overexpression via controlling gene expression using epigenetic mechanisms (specifically, by substantially blocking histone deacetylase activity), which promotes cancer cell growth arrest, differentiation, and apoptosis (Fig. 3) [63]. Valproic acid has been studied in vivo and in vitro for its possible use in various malignancies [78, 79]. Acting as an HDAC inhibitor at significantly high amounts (millimolar), valproic acid acts as a down regulator of peroxisome proliferator-activated receptors (PPAR), endorsing growth arrest, differentiation, and cell death in many types of genetic alterations of hemopoietic and non-hematopoietic origin, along with glioblastoma, melanoma, breast cancer, and lung cell lines, either alone or in combination with other chemotherapeutic agents (Fig. 3) [80, 81].

In recent years, randomized phase-2 studies have revealed that valproic acid and cytotoxic drugs show anticancer effects in hematological and solid malignancies. A post-hoc assessment of the pivotal EORT/NCI of Canada study on temozolomide. Chemoradiotherapy of patients with glioblastoma in 2011 revealed that including valproic acid contributed to a positive outcome when contrasted to the patient population with an enzyme-inducing antiepileptic drugs and those without antiepileptics drugs (Table 1) [82]. Reddy and colleagues released an article in 2014 describing the anticancer impact of valproic acid, whose concurrent administration appeared to increase the overall survival in patients with breast cancer who had brain metastasis treated with radiotherapy [83].

**Fig. 4:**
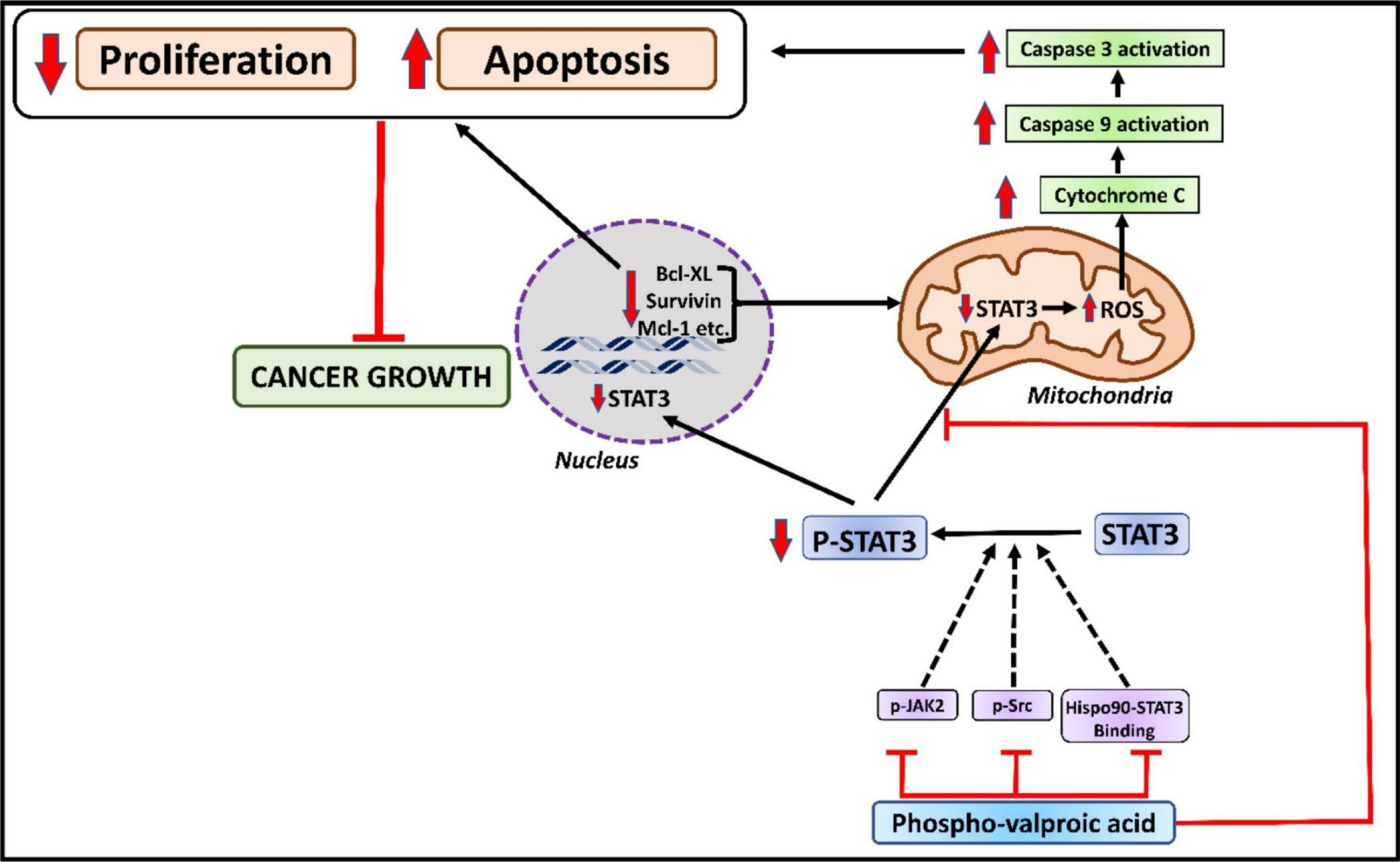
Mechanism of action of Phospho-valproic acid: An overview of the mechanism of phospho-valproic acid that blocks JAK/p-SRC/Hispo-STAT3 binding, leading to inhibition of the proliferation of STAT3 and leading downregulation of STAT3 in mitochondria. The downregulation of STAT3 leads to an increase in ROS. The generation of ROS leads to the upregulation of cytochrome c and activation of caspase 9 and caspase 3, leading to apoptosis and a decrease in proliferation, causing a decrease in cancer growth

Phospho valproic acid (demonstrated in Fig. 4), a new valproic acid derivative, was identified as a powerful and effective STAT3 inhibitor (MDC-112). This agent was created using a basic methodology in which a particular chemical alteration of known drugs improves their preferred antitumor characteristics, most notably their efficacy. Phospho-valproic acid, a branched single chain fatty acid widely used as an antiepileptic drug, is being studied for its cytotoxic activity, particularly since it has been recognized as a histone HDAC inhibitor (Fig. 4). Currently, phospho-valproic acid trials yield promising outcomes for various human cancers [84]. Clinical trial findings are encouraging, particularly those that used valproic acid with cytotoxic agents. The combination of valproic acid and epirubicin was ascertained, as was FEC100 (5-fluorouracil, epirubicin, and cyclophosphamide), an accepted regimen for breast cancer patients. In 3-week rounds, participants were given increasing amounts of valproic acid (days 1–3) and epirubicin (day 3). With tolerable toxicity, sustained plasma levels of valproic acid exceeded those needed for in vitro synergy. Furthermore, valproic acid and epirubicin were found to have anticancer activity in patients with anthracycline-resistant tumors [85].

### Oxcarbazepine

Oxcarbazepine is one of the commonly used antiepileptic drugs. Many preclinical studies have shown anticancer effects [86]. One of the studies has revealed that oxcarbazepine induces cell cycle arrest at the mitotic phase of the cell cycle. Some studies have shown that it inhibits the phosphorylation of PLK1, the main protein associated with the kinesin activation for the separation of the centrosome, cleavage of centrosomal protein, and separation of bipolar spindle assembly [86]. Other studies reveal that it inhibits HDAC, inhibiting the downstream PI3K-Akt-mTOR axis. This inhibition ultimately blocks cell proliferation and migration. This inhibition ultimately blocks mTOR and proteins Bax and Bcl-2, promoting caspase-3 and caspase9 expression and causing apoptosis and autophagy [87, 88]. Clinical investigations on the anticancer activity of oxcarbazepine are scarce and not intended to study this antiepileptic drug for antitumor action.

Furthermore, few investigations paired this drug with other enzyme-inducer antiepileptic drugs such as phenytoin, ethosuximide, primidone, phenobarbital, and carbamazepine. However, these findings are not promising. The previously stated national-wide putative Norwegian study, which recruited 1263 people with histopathological recommended glioblastoma between 2004 and 2010-half, of whom (526) were on antiepileptic drugs, found no promising effect on overall survival for the 6 examined antiepileptic drugs (valproic acid, n = 186 participants; carbamazepine, n = 163; levetiracetam, n = 195; oxcarbazepine, n = 82 and lamotrigine, n = 57).

**Fig. 5:**
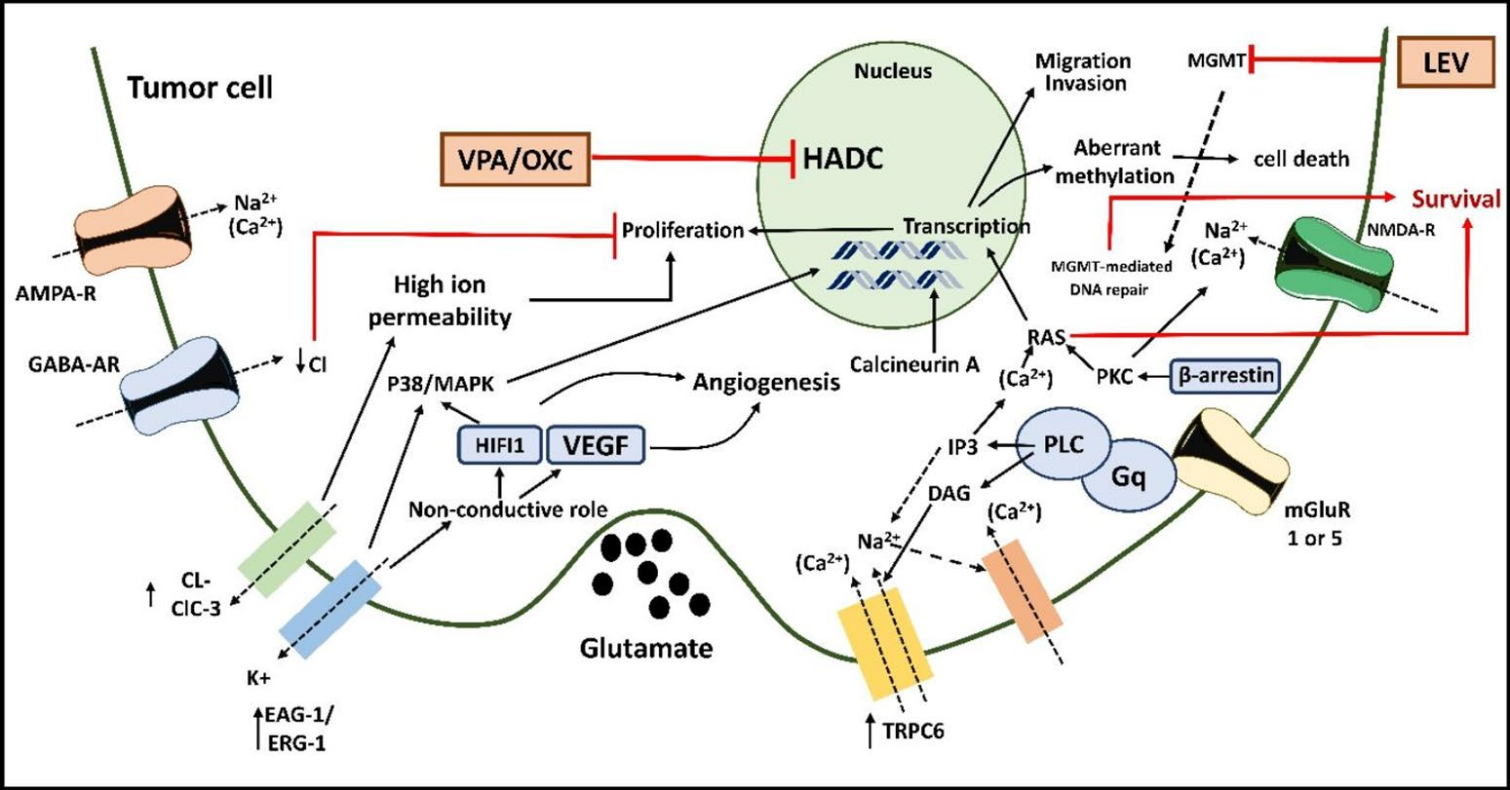
Mechanism of action of antiepileptic drugs: valproic acid (VPA) and oxcarbazepine (OXC) block the HDAC proteins leading to inhibition which blocks the downstream PTEN/PI3K-Akt axis. This inhibition ultimately blocks cell proliferation and migration. The inhibition of HDAC blocks the downstream PTEN/PI3K-Akt axis. This inhibition ultimately blocks mTOR and proteins Bax and Bcl-2, promoting caspase-3 and caspase-9 expression and causing apoptosis and autophagy. Levetiracetam acts on MGMT, preventing DNA repair mechanisms and inhibiting cancer cell survival

Figure 5 depicts the mode of action of frequently used antiepileptic drugs like oxcarbazepine, levetiracetam, and valproic acid in patients with brain tumor cells and convulsions. As demonstrated, levetiracetam, valproic acid, and oxcarbazepine-controlled seizures and inhibited the fundamental mechanisms of cell proliferation and survival. In addition to oxcarbazepine, valproic acid, and levetiracetam, other medications of choice include lamotrigine, lacosamide, zonisamide, and perampanel. In case the monotherapy is ineffective or causes adverse drug reactions, adding lacosamide due to its interaction and safety profile in brain tumor-related epilepsies (BTREs), lamotrigine due to its good safety profile and synergism with valproic acid, or zonisamide, given its latest classification as a class A medication for focal epilepsies, could be a potential therapeutic substitute (Table 1) [79, 89].

#### Antitumor effects on cell lines of different tissue origin

Amid the limited preclinical studies on oxcarbazepine’s potential anticancer activity, Cansu and colleagues’ ground-breaking study established the apoptotic and degenerative effects of rodent ovarian and uterine cells of antiepileptic drugs [87, 88]. This intriguing finding piqued the curiosity of El Sharkawi and colleagues. They investigated the antitumor effect of oxcarbazepine on three distinct solid tumor cell lines: MCF-7 (breast cancer), HepG2 (hepatocellular carcinoma), and HeLa (cervical cancer). In 2016, a parallel preliminary investigation aimed to identify the influence of antiepileptic drugs on the proliferation of glioblastoma cell lines (T98 G and U-87 MG) discovered that oxcarbazepine was considerably helpful in eliminating cell growth at therapeutic dosages, perhaps producing G2/M arrest and death (Fig. 5) [90].

.

### Lacosamide

Lacosamide belongs to 3rd-generation antiepileptic drugs that increase the delayed inactivation of voltage-gated Na^+^ channels [91]. It is regarded as an additional therapy in individuals with BTREs, capable of reducing seizure rate while causing no substantial alterations in mood or quality of life evaluations [92, 93]. The suppression of histone deacetylase is another action of lacosamide. This action may indicate that antitumor effects should be investigated. Furthermore, this mechanism has been hypothesized to explain the blockage of cell cycle migration in glioma cells, which the upregulation of miR-195-5p could cause. The same group hypothesized that by altering the transcription of other miRNAs (such as miR-107), lacosamide could decrease cellular proliferation, promote apoptotic events, and impede cell movement and invasions [94, 95]. It was demonstrated by a research group that collapsin-response-mediator-protein (CRMP2) phosphorylation (S522) was a substantial predictor of glioblastoma cellular proliferation. They used the CRMP2 phosphorylation inhibitor (S)-lacosamide to scrutinize the impact of CRMP2 phosphorylation at S522 on tumor growth and discovered that inhibiting CRMP2 phosphorylation with (S)-lacosamide reduced glioblastoma cell growth in all glioblastoma cell lines and also used showed that (S)-lacosamide inhibits glioblastoma growth in vivo models [96].

### Lamotrigine

Lamotrigine is yet another sodium-blocking antiepileptic drug. It primarily blocks voltage-gated Na^+^ channels, although it also blocks N-, L-, and P-type Calcium channels and, to a lesser extent, 5-HT3 receptors. Such effects decrease glutamate production and contribute to the stability of neuronal membranes. Lamotrigine, like lacosamide, is a suitable potential add-on medication for brain tumor individuals. According to the published research, it should be used with valproic acid, wherein the synergism can enhance the treatment of refractory epilepsies [63].

### Brivaracetam and levetiracetam

Levetiracetam was hypothesized to alter the DNA repair protein, namely MGMT, which plays a crucial function in cancer cell resistance to cytotoxic drugs like alkylating agents [97]. Levetiracetam is reported to be the potent inhibitor of MGMT among antiepileptic drugs. A multicentre, single-arm, open-label, phase-2 study was performed in Korea, where a research group showed that the principal outcome was six-month progression-free survival (PFS), and the secondary outcome was 24-month overall survival (OS) (24mo- OS). Overall survival was characterized as the time between the date of the procedure and the date of mortality from any reason. The concluding analysis included 73 patients and found that using levetiracetam during concurrent chemoradiotherapy in patients with freshly confirmed glioblastoma may contribute to enhanced effects, but more research is needed (Table 1) [98]. In vitro, studies have also shown that levetiracetam improves the efficacy of temozolomide or other anticancer drugs [99, 100].

**Table 1:** Summary of all repurposed drugs for cancer discussed in the review

Drug	Mechanism of action	Observation	Original indication	References
Valproic acid	1) Promotes GABAergic functioning 2) Decreases excitatory signals 3) Blocks histone deacetylase activity 4) Down-regulation of PPAR.	Growth arrest Cell differentiation Cell death	Antiepileptic drug	[78, 79, 82, 86–88, 92, 93, 96, 97, 99, 100]
Phospho valproic acid	1) STAT inhibition 2) HDAC inhibition	↓ Proliferation ↑ Apoptosis ↑ Caspase 3	Antiepileptic drug	
Oxcarbazepine	HDAC inhibition	↓ Cell growth Anti-proliferative Apoptosis	Antiepileptic drug	
Lacosamide	1) ↑delayed inactivation of Na^+^ channel 2) HDAC inhibition	↓ Cell growth Apoptosis	Antiepileptic drug	
Lamotrigine	Inhibition of Na^+^channel	Apoptosis Anti-proliferative	Antiepileptic drug	
Brivaracetam and levetiracetam	MGMT inhibition	↑ OS. ↑ Cell death	Antiepileptic drug	

## Conclusions

To our understanding, this evaluation uncovers that certain anticonvulsants are beneficial in blocking tumor cell proliferation and expansion. Clinical evidence on potential antiepileptic drug influence is still limited, and numerous contributing factors have been linked to the research findings. Clinical data show unsatisfactory results in classifying anticonvulsants as antineoplastic drugs. Nevertheless, even though the necessity to construct and conduct potential clinical tests concentrated on the cytotoxic activity of antiepileptic drugs, and that can obtain a significant amount of people pre-emptively selected as per stringent and appropriate criteria for inclusion, persists to be an expansive concern, the current review provided evidence and statistics which may encourage the experimentation of novel pharmacological techniques for the cancer treatment, such as PK/PD designs and computational decision-making methods.
